# A Genome Wide Study of Copy Number Variation Associated with Nasopharyngeal Carcinoma in Malaysian Chinese Identifies CNVs at 11q14.3 and 6p21.3 as Candidate Loci

**DOI:** 10.1371/journal.pone.0145774

**Published:** 2016-01-05

**Authors:** Joyce Siew Yong Low, Yoon Ming Chin, Taisei Mushiroda, Michiaki Kubo, Gopala Krishnan Govindasamy, Kin Choo Pua, Yoke Yeow Yap, Lee Fah Yap, Selva Kumar Subramaniam, Cheng Ai Ong, Tee Yong Tan, Alan Soo Beng Khoo, The Malaysian NPC Study Group, Ching Ching Ng

**Affiliations:** 1 Institute of Biological Sciences, Faculty of Science, University of Malaya, Kuala Lumpur, Malaysia; 2 Translational Genomics Lab, High Impact Research Building (Level 2), University of Malaya, Kuala Lumpur, Malaysia; 3 Laboratory for Pharmacogenetics, RIKEN Center for Integrative Medical Sciences, Yokohama, Japan; 4 Laboratory for Genotyping Development, RIKEN Center for Integrative Medical Sciences, Yokohama, Japan; 5 Department of Otorhinolaryngology, Faculty of Medicine, University of Malaya, Kuala Lumpur, Malaysia; 6 Department of Otorhinolaryngology, Hospital Pulau Pinang, Penang, Malaysia; 7 Department of Surgery, Faculty of Medicine and Health Sciences, Universiti Putra Malaysia, Kuala Lumpur, Malaysia; 8 Department of Oral Biology & Biomedical Sciences and Oral Cancer Research & Coordinating Centre, Faculty of Dentistry, University of Malaya, Kuala Lumpur, Malaysia; 9 Department of Otorhinolaryngology, Head and Neck Surgery, Sarawak General Hospital, Sarawak, Malaysia; 10 ENT Department, Hospital Queen Elizabeth, Karung Berkunci No. 2029, Kota Kinabalu, Sabah, Malaysia; 11 Department of Otorhinolaryngology, Sarawak General Hospital, Kuching, Sarawak, Malaysia; 12 Molecular Pathology Unit, Cancer Research Centre, Institute for Medical Research, Kuala Lumpur, Malaysia; 13 The Malaysian Nasopharyngeal Carcinoma Study Group: Hospital Pulau Pinang, Hospital Kuala Lumpur/Universiti Putra Malaysia, University of Malaya, Institute for Medical Research, Cancer Research Initiatives Foundation, Sarawak General Hospital/Universiti Malaysia Sarawak, Queen Elizabeth Hospital and Hospital Universiti Sains, Malaysia; The Chinese University of Hong Kong, HONG KONG

## Abstract

**Background:**

Nasopharyngeal carcinoma (NPC) is a neoplasm of the epithelial lining of the nasopharynx. Despite various reports linking genomic variants to NPC predisposition, very few reports were done on copy number variations (CNV). CNV is an inherent structural variation that has been found to be involved in cancer predisposition.

**Methods:**

A discovery cohort of Malaysian Chinese descent (NPC patients, n = 140; Healthy controls, n = 256) were genotyped using Illumina^®^ HumanOmniExpress BeadChip. PennCNV and cnvPartition calling algorithms were applied for CNV calling. Taqman CNV assays and digital PCR were used to validate CNV calls and replicate candidate copy number variant region (CNVR) associations in a follow-up Malaysian Chinese (NPC cases, n = 465; and Healthy controls, n = 677) and Malay cohort (NPC cases, n = 114; Healthy controls, n = 124).

**Results:**

Six putative CNVRs overlapping *GRM5*, *MICA/HCP5/HCG26*, *LILRB3/LILRA6*, *DPY19L2*, *RNase3/RNase2* and *GOLPH3* genes were jointly identified by PennCNV and cnvPartition. CNVs overlapping *GRM5* and *MICA/HCP5/HCG26* were subjected to further validation by Taqman CNV assays and digital PCR. Combined analysis in Malaysian Chinese cohort revealed a strong association at CNVR on chromosome 11q14.3 (*P*_combined_ = 1.54x10^-5^; odds ratio (OR) = 7.27; 95% CI = 2.96–17.88) overlapping *GRM5* and a suggestive association at CNVR on chromosome 6p21.3 (*P*_combined_ = 1.29x10^-3^; OR = 4.21; 95% CI = 1.75–10.11) overlapping *MICA/HCP5/HCG26* genes.

**Conclusion:**

Our results demonstrated the association of CNVs towards NPC susceptibility, implicating a possible role of CNVs in NPC development.

## Introduction

Nasopharyngeal carcinoma (NPC) (OMIM 161550 and 607107) is a malignant tumour arising in the nasopharyngeal mucosa. NPC displays a geographic bias. It is a rare malignancy in most parts of the world (ASR = 1 case per 100,000 per year), but a highly endemic disease in selected demographics such as Southern Chinese population of Guangdong (as high as 15 to 25 cases per 100,000 per year) [1], native Greenlanders [2] and migrants of the Southern Chinese in America, Australia, and Malaysia [3, 4]. The skewed demographic distribution of the disease coupled with familial aggregation [5], Epstein-Barr virus (EBV) infection and environmental factors such as air pollutants [6], dietary intake of salted fish and preserved food [7], suggest possible interplay between genetic and environmental factors in NPC pathogenesis. Many genetic studies on single nucleotide polymorphisms (SNPs) have reported associations to NPC. Genes linked to NPC include *HLA-A* (Human leukocyte antigen A) [8, 9], *HLA-B* (Human leukocyte antigen B) [10], *GABBR1* (Gamma-aminobutyric acid (GABA) B receptor 1) [11], *CYP2E1* (cytochrome P450 2E1) and *hOGG1* (human 8-oxoguanine DNA N-glycosylase 1) [12]. Despite many reports linking SNP variants to NPC predisposition, structural variants such as CNVs and its possible influence on NPC predisposition remain grossly neglected. Thus far, only deletions linked to NPC have been reported at chromosome 3p [13] and 6p21.3 [14].

Copy number variants are structural variants present in multiple copies involving DNA segments of ≥ 1 kb [15]. CNVs can have simple structures such as tandem duplication or complex structures like sequence complexity at junctions that arise from complex amplification and deletion rearrangements. Some CNVs remain neutral in function, others convey differences in phenotypes by disrupting genes, creating fusion genes, changing copy number of dosage sensitive genes and altering the regulatory transcription levels [16]. These changes in phenotypes can be benign or can have detrimental implications in diseases such as osteoporosis [17], schizophrenia [18], Li-Fraumeni-syndrome-associated cancers [19] and neuroblastoma [20].

The Malaysian Chinese population is largely made up of descendants from Southern China. This population comprises various dialect groups of the Hokkien, Hakka, Cantonese, Teochew and Hainanese. NPC incidence is high among the Malaysian Chinese, especially in Chinese males (ASR = 14 per 100,000) [21]. As for the Malaysian Malay population, they are a heterogeneous ethnic group with different ancestral origins based on migration patterns of centuries ago. They were the descendants of the Proto-Malays, admixed with Siamese, Javanese, Sumatran, Indian, Thai, Arab and Chinese traders [22]. The Malays make up the majority of the Malaysian population. NPC incidence is low among the Malays, including the Malay males (ASR = 4.0 per 100,000) [21].

This study aims to evaluate the role of CNVs in NPC predisposition in the Malaysian Chinese and Malay populations. We report a case control genome-wide SNP microarray approach to identify CNVs associated to NPC in the Malaysian Chinese and Malay populations.

## Materials and Methods

### Study samples

Samples were recruited from the University Malaya Medical Centre (UMMC), Penang General Hospital (HPP), Kuala Lumpur General Hospital (HKL), Sarawak General Hospital (HUS) and Queen Elizabeth Hospital Sabah (QES) from year 2006 to 2013. All cases were histo-pathologically diagnosed according to the World Health Organization (WHO) classification. GWAS discovery cohort consisted of 193 cases and 290 controls (140 cases and 256 healthy controls after QC filtering) of Malaysian Chinese descent and the replication cohort consisted of 465 NPC patients and 677 healthy controls of Malaysian Chinese descent, giving a combined cohort of 605 cases and 933 controls after QC filtering. The Malaysian Malay replication cohort consisted of 114 cases and 124 controls of Malaysian Malay descent. Demographic characteristics of Malaysian Chinese study participants are described in S1 Table and demographic characteristics of Malaysian Malay study participants are described in S2 Table.

### Ethical approval and consent

All cases and controls gave written informed consent and the study was approved by Medical Research Ethic Committee (MREC) Ministry of Health Malaysia (Registration ID: NMRR-13-969-17878), ethical committees of the Yokohama Institute, The Institutes of Physical and Chemical Research (RIKEN), Yokohama, Japan and Medical Ethics Committee of University Malaya Medical Centre.

### Genotyping, quality control and correction for population structure

Genome-wide genotyping of NPC cases and healthy controls of Malaysian Chinese was conducted using Illumina^®^ HumanOmniExpress_12 v1.1 Genotyping BeadChip (San Diego, CA, USA) according to manufacturer's protocol. The genomic DNA was extracted from peripheral blood leukocytes using conventional phenol-chloroform method. Samples with sample call rates <0.99, mismatch between recorded and estimated gender, cryptic relatedness and population outliers were excluded from the subsequent studies. Population outliers were identified using Principal component analysis (PCA) performed in EIGENSTRAT version 2.0 [23].

### Generation of CNV calls

Samples passing quality control measures were used for generating CNV calls. Log R ratio (LRR) and B allele frequency (BAF) were generated from intensity data of the genome-wide genotyping using GenomeStudio (v.3.1.6) (San Diego, CA, USA) and CNVs were called using PennCNV (v.2011 Jun16) [24] and cnvPartition (v.3.1.6) (San Diego, CA, USA). For uniformity, samples with standard deviation (SD) of LRR≥ 0.20, SD of BAF ≥ 0.2, BAF drifting value of ≥ 0.01, waviness factor ≥ 0.05 and samples with more than 50 CNV calls were removed from analysis using PennCNV prior to CNV calling. Centromeric regions, telomeric regions, XY chromosomes and regions coding for immunoglobulin genes were also excluded from CNV calling. Minimum number of markers of 5 were set as threshold for all CNV calling. For cnvPartition analysis, confidence score threshold was set to 35 and minimum number of probes was set to 5.

### CNV validation and replication

Validation of GWAS CNV calls and replication of CNV association at candidate loci on chromosome 11q14.3 and chromosome 6p21.3 were performed using Taqman CNV genotyping assays (ABI, Foster City, CA) on the GWAS discovery cohort (Malaysian Chinese only) and replication cohort (Malaysian Chinese and Malaysian Malay). Quantification cycle (C_q_) of each region of interest was determined by QuantStudio^™^ 12K Flex Software (v.1.0, Applied Biosystem, Foster City, CA) and copy numbers were determined using Copy Caller (v.2.0, Applied Biosystems, Foster City, CA) according to a comparative delta Cq method. Random samples with a mixture of copy number (CN) state of 1, 2 and 3 were subjected to validation with Droplet digital PCR (ddPCR) (Bio-Rad Laboratories, Hemel Hempstead, UK) following manufacturer’s protocol.

### Gene-set enrichment analysis

Five pathways previously found to be associated to NPC were subjected to enrichment analysis [25]. Enrichment analysis was carried out using cnv-enrichment-test [26] implemented in PLINK v.1.07 [27]. Gene sets were compiled from the Molecular Signatures Database (MSigDB) v.3.1 namely KEGG WNT signalling pathway, ST ERK1 ERK2 MAPK pathway, KEGG NOTCH signalling pathway, REACTOME regulation of apoptosis and REACTOME signalling by EGFR in cancer. One sided empirical *P*-values with 10,000 permutation of enrichment of a subset of genes of a pathway relative to all genes were acquired.

### Global CNV burden analysis

PLINK v.1.07 [27] was used to perform global CNV burden analysis. CNVs were classified as rare when found in <1% of total GWAS samples or common when found in ≥ 5% of total GWAS samples. Tests for CNV burden (one-sided) were done for number of CNV segments per individual, number of genes overlapped by CNVs per individual and number of genes intersected per total CNV kb using UCSC RefSeq (hg18) gene annotation. CNVs were considered co-localized if they overlapped by at least 50% of their length.

### HLA allele imputation

SNPs that pass quality control filtering on chromosome 6 were input into the SNP2HLA software [28] in forms of PLINK formats of (.bed/.bim/.fam). SNP allele annotations were mapped to forward strand and physical coordinates corresponding to hg18. Default parameter settings for SNP2HLA were used in the phasing and imputation. The Pan-Asian reference panel was used for this study [29, 30].

### Statistical association analysis

Copy number variant regions (CNVRs) are identified using reciprocal overlap (minimum threshold 50%). Logistic regression using PLINK v.1.07 was carried out to assess the frequency difference between NPC cases and healthy controls using gender and age as covariates. Gene-based association was carried out using ParseCNV v.17 [31]. GWAMA v.1.4 [32] was used for *I*^*2*^ heterogeneity analysis. G*Power v.3.1.9.2 was employed for power analysis for z tests logistic regression, using post-hoc computation of achieved power, one tail at α = 0.05 setting [33].

## Results

### Genome-wide CNV Distribution in the Malaysian Chinese GWA Sample

**Fig 1:**
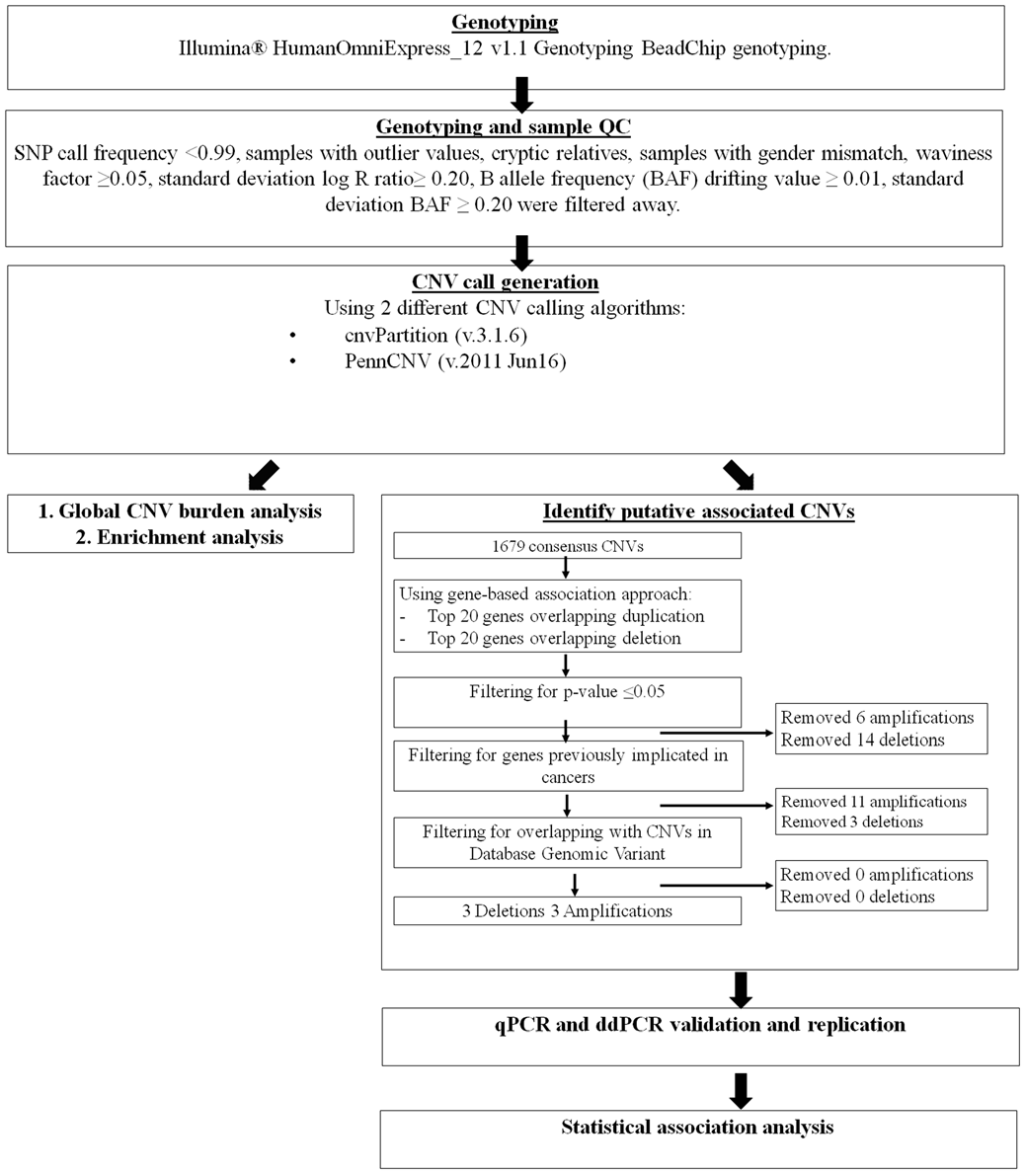
Flowchart illustrating workflow for whole genome CNV analysis and NPC susceptibility associated candidate gene selection from our GWAS discovery cohort.

Our CNV analysis workflow is detailed in Fig 1. From the 733,202 genotyped SNPs, 13,365 SNPs were removed due to low call frequency (<0.99). In total, 9 cases and 24 controls were removed due to population outliers, mismatch between recorded and estimated gender and cryptic relatedness. CNV calling quality control measures removed a further 44 cases and 13 controls due to high standard deviation of Log R ratio (SD BAF ≥ 0.2), excessive CNV calls (≥50 CNVs) and drifting B allele frequency values (BAF drifting value of ≥ 0.01). Post quality control measures, 140 cases and 256 controls remained and were used for downstream CNV analysis.

For global CNV analysis, we used only stringent consensus CNVs from both PennCNV and cnvPartition calling algorithms. Total of 1679 CNVs were called (mean size = 147,798bp; median size = 66739bp; range size = 720bp- 4,979,575bp). CNV burden analysis revealed no significant difference in global distribution of rare CNVs between cases and controls (Table 1). There was also no significant difference in common CNV rates (CNV_freq_≥5%) (*P* = 0.5326) (Table 1), although common CNVs were 5.62 times more enriched with genes in cases than in controls (*P* = 7.00x10^-4^) (Table 1). NPC cases also had an over-representation of common genic deletions compared to controls (Case/control ratio = 2.26, *P* = 0.03). There were however no significant difference between cases and controls in terms of rare CNV (CNV_freq_<1%) rate and gene enrichment.

**Table 1:** Global CNV burden in NPC patients and healthy controls.

	Rate^a^		Gene count (GRate)^b^		Gene count (GRich)^c^	
	Case/control ratio	*P*-value^d^	Case/control ratio	*P*-value^d^	Case/control ratio	*P*-value^d^
Types						
Deletion	0.8421	0.9432	0.9967	0.5112	2.2589	0.0310*
Amplification	1.2225	0.0029**	0.9426	0.677	1.3646	0.2043
Size						
<100kb						
Deletion	0.8849	0.9945	1.2726	0.8394	2.8430	0.0085**
Amplification	1.3108	0.0022**	1.2394	0.0816	1.2736	0.1975
≥100kb						
Deletion	0.7314	0.9874	0.8658	0.7288	1.0636	0.3843
Amplification	1.1217	0.1739	0.8587	0.8292	1.1051	0.2074
Frequency						
Rare	0.9120	0.8867	0.9924	0.5443	1.5081	0.1318
Common	0.9967	0.5326	3.9612	4x10^-05^***	5.6200	0.0007***

^a^Rate: Number of CNVs per individual

^b^Gene count (GRate): Number of genes spanned by CNVs per individual

^c^Gene count (GRich): Number of genes per total CNV kb

^d^*P*-value: Based on comparison between cases and controls, one-sided, permuted 100,000 times

*0.01<*P*-value<0.05

**10^−04^<*P*-value<0.01

***P-value<10^−04^

### CNVRs associated to NPC susceptibility and pathway enrichment

**Fig 2:**
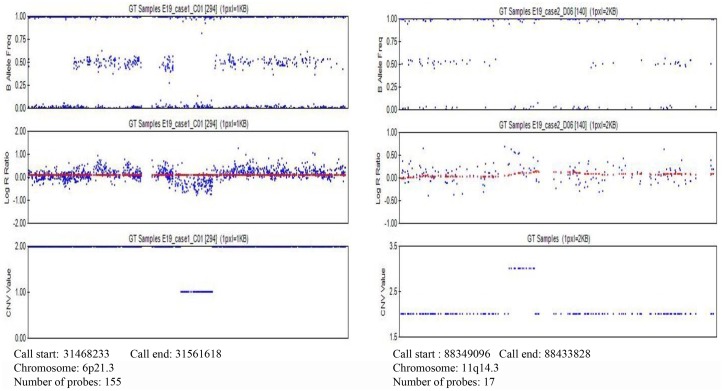
An illustration of B allele frequency, Log R ratio, and CNV value for CNVs at chromosome 6p21.3 (left) and 11q14.3 (right) generated from GenomeStudio (v.3.1.6).

We focused on consensus CNVs called from both PennCNV and cnvPartition calling algorithms. From the consensus CNVRs, we selected regions overlapping genes and genomic regions previously linked to NPC and other cancers. We also checked for genes differentially expressed in NPC tissue specimens compared to normal nasopharyngeal tissue [34, 35]. Six candidate CNVRs fulfilling the selection criteria were presented in Table 2. Further qPCR validation was done on CNVR 11q14.3 and 6p21.3 and Fig 2 provided showed example LRR and BAF in these CNVs. LRR and BAF from SNP data within CNVs in chromosome 6 (S1 Text) and chromosome 11 (S2 Text) can be found in the supporting information. Concordance rate between CNV calls generated from calling algorithms and qPCR was shown in Table 3. As shown in Table 3, PennCNV calls had higher concordance for both CNVRs on chromosome 6p21.3 (99.75%) and 11q14.3 (91.67%). Taqman genotyping of CNVRs 11q14.3 and 6p21.3 confirmed suggestive association in the Malaysian Chinese discovery cohort (*P*_11q14.3_Discovery_ = 4.60x10^-3^; *P*_6p21.3 Discovery_ = 0.051) (Table 4). CNVRs 11q14.3 and 6p21.3 were selected for further replication in a separate Malaysian Chinese (465 cases and 677 controls) and Malaysian Malay (114 cases and 124 controls) replication cohort.

**Table 2:** List of most significantly associated candidate CNVRs identified from GWAS cohort.

Cytoband	Risk allele	Intersecting genes	Potential function in cancer	CNV from Database of Genomic Variants	Previous studies (Association to cancer/ NPC)	Previous studies (Differential expression in NPC tissue)^a^
Chromosome 11q14.3	Amp	*GRM5*	Glutamatergic signalling has been shown to stimulate proliferation and migration of tumour cell lines.[42]	Yes	- Implicated in melanoma,[42] oral squamous cell carcinoma[43]	Upregulated
Chromosome 6p21.3	Del	*MICA*, *HCP5*, *HCG26*	Cytolytic responses of T cells and NK cells against MICA-expressing tumour cells were activated when NKG2D binds with MICA.[51]	Yes	- CNV association with several NPC study cohort[14, 47]	No differential expression for all genes.
Chromosome 19q13.42	Del	*LILRB3*, *LILRA6*	B cell receptor signalling.	Yes	- CNV associated to NPC in NPC Taiwan genome-wide study cohort[14]	No differential expression
Chromosome 12q14.2	Amp	*DPY19L2*	Unknown.	Yes	- Loss of heterozygosity associated with lung adenocarcinoma[52]	No differential expression
Chromosome 5p13.3	Amp	*GOLPH3*	Vesicular trafficking.	Yes	- Associated with various cancers e.g. lung cancer,[53] prostate cancer,[54] gastric cancer [55]	No differential expression
Chromosome 14q11.2	Del	*RNase3*, *RNase2*	Ribonuclease family	Yes	- Associated with pancreatic cancer[56]	Downregulated

Amp (Amplification); Del (Deletion); *GRM5* (Metabotropic glutamate receptor 5); *MICA* (MHC class I polypeptide-related sequence A); *HCP5* (HLA complex 5); *HCG26* (HLA complex group 26); *LILRB3* (Leukocyte immunoglobulin-like receptor subfamily B member 3); *LILRA6* (Leukocyte immunoglobulin-like receptor subfamily A member 6; *DPY19L2* (Dpy-19-like protein 2); *GOLPH3* (Golgi phosphoprotein 3); *RNase3* (Ribonuclease, RNase A family, 3); *RNase2* (Ribonuclease, RNase A family, 2).

^a^ Differential expression data was extracted from Dodd *et al*.[34] MSigDB v4.0[57] curated gene sets.

**Table 3:** Results from multiple CNV calling algorithms and qPCR validation of CNVRs on chromosome 6p21.3 and chromosome 11q14.3.

Cytoband	Risk allele	CNV calling algorithm	CNVR boundaries	Concordance rate with qPCR	Genes overlapped
Chr 6p21.3	CN = 1	PennCNV	Chr6:31468233–31562343	99.75%	*MICA*, *HCP5*, *HCG26*
		cnvPartition	Chr6:31468233–31571696	99.50%	
Chr 11q14.3	CN = 3	PennCNV	Chr11:88309000–88433828	91.67%	*GRM5*
		cnvPartition	Chr11:88336310–88433828	90.15%	

*MICA* (MHC class I polypeptide-related sequence A); *HCP5* (HLA complex 5); *HCG26* (HLA complex group 26); *GRM5* (Metabotropic glutamate receptor 5)

**Table 4:** Association of CNVRs on chromosome11q14.3 and chromosome 6p21.3 with susceptibility in discovery and validation cohorts of Malaysian Chinese.

CNVR genomic position	Discovery cohort^a^				Malaysian Chinese replication cohort				Combined	
	Case (%) n = 140	Control (%) n = 256	*P*-value	OR (95% CI)	Case (%) n = 465	Control (%) n = 677	*P*-value	OR (95% CI)	*P*-value	OR (95% CI)
Chr6p21.3	7 (5.00)	3 (1.17)	0.051	5.84 (0.99–34.49)	14 (3.00)	8 (1.18)	0.011	3.74 (1.36–10.31)	1.29x10^-3^	4.21 (1.75–10.11)
Chr11q14.3	15 (10.71)	1 (0.39)	4.6x10^-3^	27.59 (2.78–274.50)	20 (4.2)	8 (1.18)	2.6x10^-3^	4.79 (1.73–13.30)	1.54x10^-5^	7.27 (2.96–17.88)

^a^ Results are based on CNV calls from genome-wide genotyping data after validation with qPCR.

**Fig 3:**
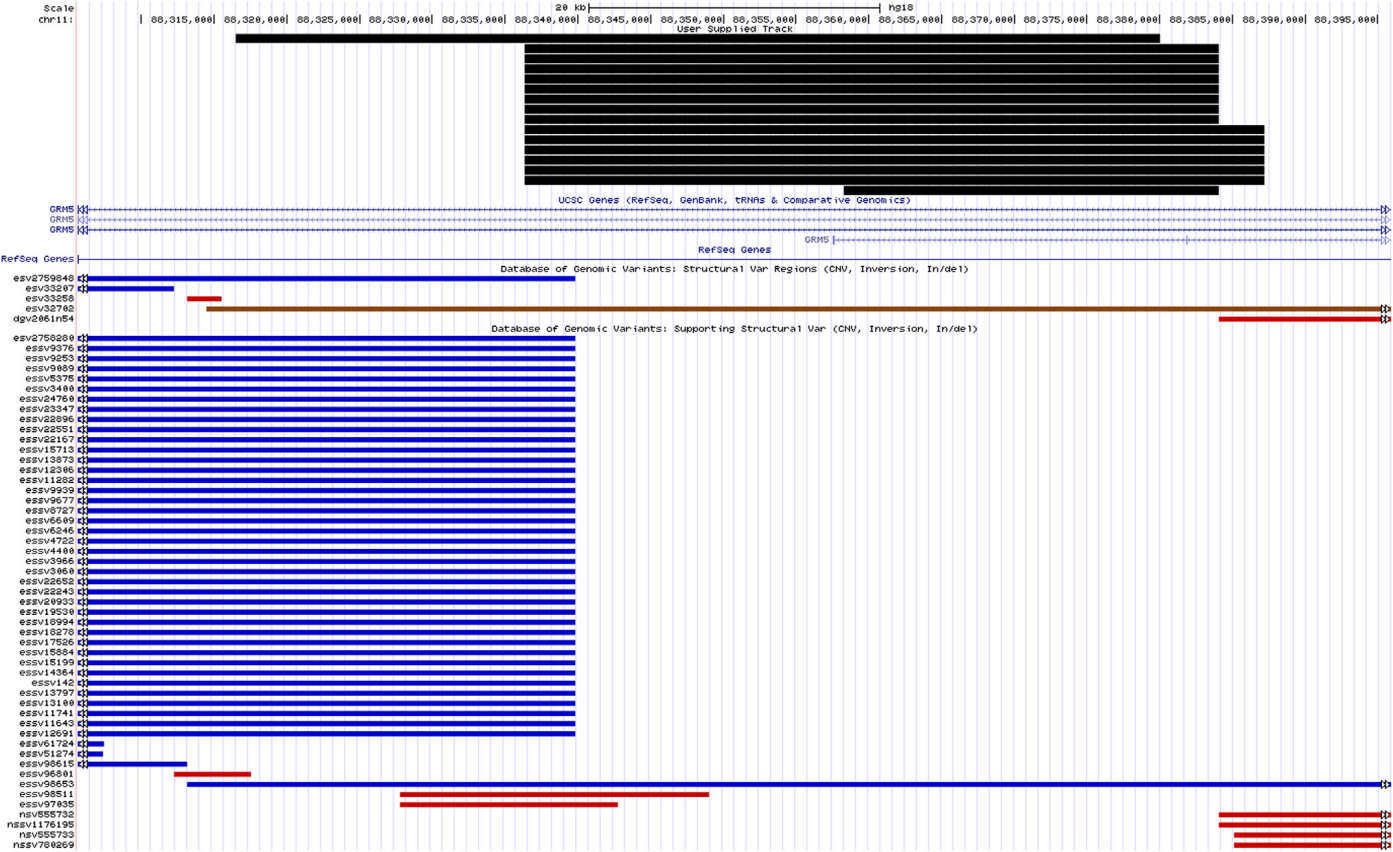
Schematic representation of the genomic organization according to the human genome hg18 at chromosome 11q14.3 with the positions of CNVs called in the region and reported CNVs in the vicinity found in the Database of Genomic Variants, accessed by July 2015. Blue bars represent gains identified by other studies that were deposited in the database while red bars represent losses. Brown bars depict CNVs with both losses and gains documented.

From our combined analysis, CNVR on chromosome 11q14.3 showed the strongest association to NPC susceptibility in our Malaysian Chinese cohort (*P*_combined_ = 1.54x10^-5^; OR = 7.27; 95% CI = 2.96–17.88) (Table 4). In our study, the most common recurrent CNVs at chromosome 11q14.3 were hg18 chr11:88,336,310–88,384,073 (8 cases) and hg18 chr11: 88,336,310–88,387,195 (6 cases) (Fig 3). CNVR 11q14.3 was however not associated to NPC susceptibility in the Malaysian Malay cohort (*P* = 0.99; OR = 10.14; 95% CI = 0.54–190.47) (Table 5). Heterogeneity test done between discovery and Malaysian Chinese validation cohorts using GWAMA v.1.4 provided *I*^*2*^ value of 0.7. *I*^*2*^ value was 0.7 between discovery and validation cohort of Malaysian Chinese, however the association of CNV 11q14.3 for both discovery (p-value = 4.6x10^-3^) and validation cohorts (p-value = 2.60x10^-3^) were significant and consistent in direction.

**Table 5:** Association of CNVRs on chromosome11q14.3 and chromosome 6q21.33 with susceptibility in Malaysian Malay replication cohort.

CNVR genomic position	Malaysian Malay replication cohort			
	Case (%) n = 114	Control (%) n = 124	*P*-value	OR (95% CI)
Chr6p21.3	6 (5.26)	4 (3.23)	0.32	2.10 (0.48–9.28)
Chr11q14.3	4 (3.51)	0 (0)	0.99	10.14 (0.54–190.47)

**Fig 4:**
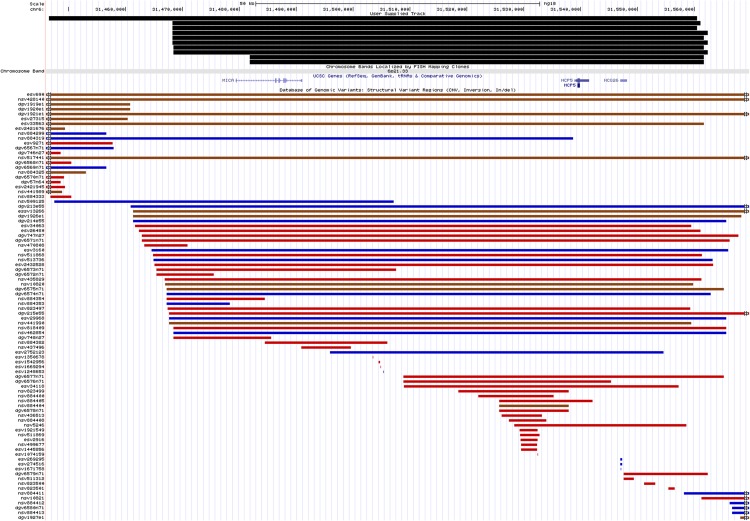
Schematic representation of the genomic organization according to the human genome hg18 at chromosome 6p21.3 with the positions of CNVs called in the region and reported CNVs in the vicinity found in the Database of Genomic Variants, accessed by July 2015. Blue bars represent gains identified by other studies that were deposited in the database while red bars represent losses. Brown bars depict CNVs with both losses and gains documented.

The combined association of CNVR on chromosome 6p21.3 was more modest in the Malaysian Chinese cohort (*P*_combined_ = 1.29x10^-3^; OR = 4.21; 95% CI = 1.75–10.11) (Table 4) (Fig 4). From our results, we observed that carriers of the 6p21.3 deletion also carried the HLA-B*4801 (8/10) or HLA-C*0801 (8/10) allele (Table 6). CNVR 6p21.3 was not associated with NPC susceptibility in the Malay cohort (*P* = 0.32) (Table 5).

**Table 6:** *HLA-A*, *HLA-B*, and HLA-C alleles of samples with heterozygous deleted *MICA* from HLA imputation software SNP2HLA.

Sample ID	*MICA* copy number	HLA-A allele	HLA-B allele	HLA-C allele
E19_case1_C01	1	0201/1101	4001/4801	0304/0702
E19_case1_C09	1	1101/1101	1301/1502	0801/0801
E19_case1_D04	1	0207/2402	4601/4801	0102/0801
E19_case1_D08	1	0206/2402	1501/4801	0401/0801
E19_case1_E11	1	0203/2402	4801/5101	0801/1402
E19_case2_D07	1	1101/2402	4002/4801	0702/0801
E19_case2_G03	1	0201/0207	4601/4801	0102/0801
E19_cont2_G01	1	1101/2601	3901/4801	0702/0801
E19_cont2_G03	1	0201/0203	4001/4601	0102/0702
E19_cont3_D10	1	0206/1101	4801/5401	0102/0801

The CNVR 11q14.3 and 6p21.3 copy numbers identified through Taqman qPCR were highly concordant with digital PCR copy numbers though comparison of CNV copy numbers was limited to only 10 random samples.

**Fig 5:**
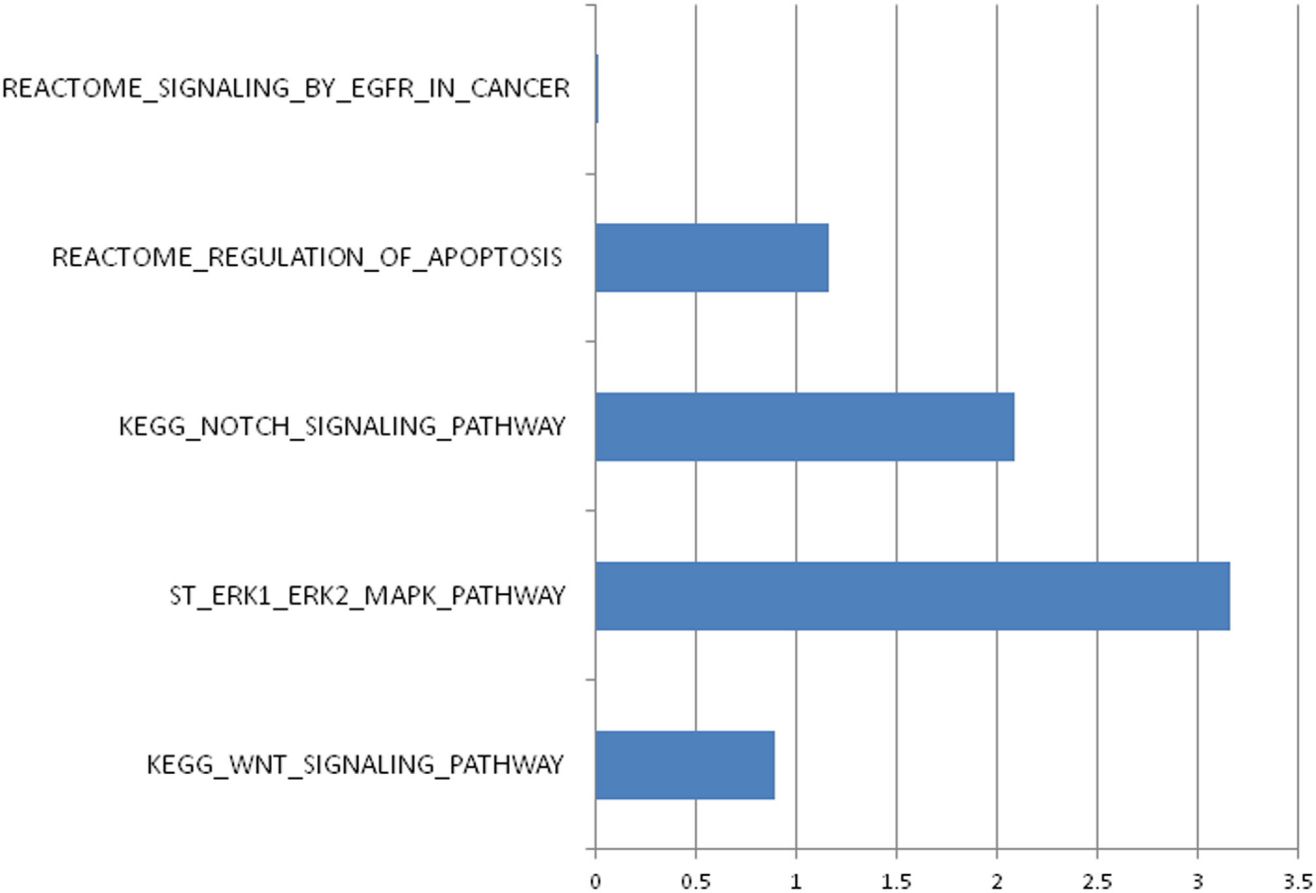
-Log_10_ of one-tailed empirical p-values from pathway enrichment analysis implemented using PLINK v.1.07 *cnv-enrichment-test*.

The CNVs were subjected to pathway enrichment using a gene set-enrichment method [26] implemented in PLINK v1.07. The ERK1/ERK2-MAPK pathway and NOTCH Signaling pathway were identified and deemed to be significantly enriched (*P*_ERK-MAPK_ = 7.00x10^-4^; P_Notch_ = 8.30x10^-3^) (Fig 5) (Table 7).

**Table 7:** p-values of gene-set enrichment analysis for five pathways previously associated to nasopharyngeal carcinoma.

Pathway	Genes	p-value
KEGG WNT signalling pathway	*JUN*, *LRP5*, *LRP6*, *PPP3R2*, *SFRP2*, *SFRP1*, *PPP3CC*, *VANGL1*, *PPP3R1*, *FZD1*, *FZD4*, *APC2*, *FZD6*, *FZD7*, *SENP2*, *FZD8*, *LEF1*, *CREBBP*, *FZD9*, *PRICKLE1*, *CTBP2*, *ROCK1*, *CTBP1*, *WNT9B*, *WNT9A*, *CTNNBIP1*, *DAAM2*, *TBL1XR1*, *MMP7*, *CER1*, *MAP3K7*, *VANGL2*, *WNT2B*, *WNT11*, *WNT10B*, *DKK2*, *LOC728622*, *CHP2*, *AXIN1*, *AXIN2*, *DKK4*, *NFAT5*, *MYC*, *SOX17*, *CSNK2A1*, *CSNK2A2*, *NFATC4*, *CSNK1A1*, *NFATC3*, *CSNK1E*, *BTRC*, *PRKX*, *SKP1*, *FBXW11*, *RBX1*, *CSNK2B*, *SIAH1*, *TBL1Y*, *WNT5B*, *CCND1*, *CAMK2A*, *NLK*, *CAMK2B*, *CAMK2D*, *CAMK2G*, *PRKACA*, *APC*, *PRKACB*, *PRKACG*, *WNT16*, *DAAM1*, *CHD8*, *FRAT1*, *CACYBP*, *CCND2*, *NFATC2*, *NFATC1*, *CCND3*, *PLCB2*, *PLCB1*, *CSNK1A1L*, *PRKCB*, *PLCB3*, *PRKCA*, *PLCB4*, *WIF1*, *PRICKLE2*, *PORCN*, *RHOA*, *FRAT2*, *PRKCG*, *MAPK9*, *MAPK10*, *WNT3A*, *DVL3*, *RAC2*, *DVL2*, *RAC3*, *FZD3*, *DKK1*, *CXXC4*, *DVL1*, *FOSL1*, *CUL1*, *WNT10A*, *WNT4*, *SMAD3*, *TCF7*, *SMAD4*, *RAC1*, *TCF7L2*, *SMAD2*, *WNT1*, *MAPK8*, *EP300*, *WNT7A*, *GSK3B*, *WNT7B*, *PSEN1*, *WNT8A*, *WNT8B*, *WNT2*, *WNT3*, *WNT5A*, *WNT6*, *CTNNB1*, *PPP2CB*, *PPP2CA*, *PPP2R1A*, *TBL1X*, *PPP2R1B*, *ROCK2*, *NKD1*, *FZD10*, *FZD5*, *NKD2*, *TCF7L1*, *RUVBL1*, *PPARD*, *PPP3CB*, *TP53*, *PPP3CA*, *PPP2R5A*, *PPP2R5E*, *PPP2R5D*, *PPP2R5C*, *PPP2R5B*, *FZD2*, *SFRP5*, *SFRP4*, *CHP*	0.1283
ST ERK1 ERK2 MAPK pathway	*SOS1*, *RPS6KA3*, *TRAF3*, *CREB3*, *MAPK3*, *EIF4E*, *SOS2*, *MAP3K8*, *RPS6KA2*, *EEF2K*, *CREB5*, *MAP2K1*, *SHC1*, *KLF6*, *RAP1A*, *ARAF*, *DUSP4*, *MKNK1*, *GRB2*, *DUSP9*, *BRAF*, *KAT5*, *MKNK2*, *BAD*, *MAP2K2*, *ATF1*, *NFKB1*, *CREB1*, *RPS6KA1*, *MAPK1*, *MOS*, *DUSP6*	0.0007
KEGG NOTCH signalling pathway	*HES5*, *DTX3*, *NOTCH4*, *DTX3L*, *NOTCH3*, *NOTCH2*, *EP300*, *HES1*, *NOTCH1*, *NUMB*, *PSEN2*, *PSEN1*, *PTCRA*,*SNW1*, *APH1A*, *KAT2A*, *ADAM17*, *RFNG*, *RBPJ*, *DTX1*, *CREBBP*, *DTX2*, *MAML1*, *CTBP2*, *NCOR2*, *CTBP1*, *DVL3*, *JAG2*, *DVL2*, *NUMBL*, *MAML2*, *KAT2B*, *DLL4*, *PSENEN*, *DLL3*, *DVL1*, *CIR1*, *DLL1*, *LFNG*, *JAG1*, *MAML3*, *HDAC1*, *HDAC2*, *NCSTN*, *DTX4*, *MFNG*, *RBPJL*	0.0083
REACTOME regulation of apoptosis	*PSMD14*, *PSMA8*, *DAPK1*, *DAPK3*, *DCC*, *UNC5B*, *PSME4*, *DAPK2*, *APPL1*, *PAK2*, *PSMA1*, *PSMA2*, *PSMA3*, *PSMA4*, *PSMA5*, *PSMA6*, *PSMA7*, *PSMB1*, *PSMB2*, *PSMB3*, *PSMB4*, *PSMB5*, *PSMB6*, *PSMB7*, *PSMB8*, *PSMB9*, *PSMB10*, *PSMC1*, *PSMC2*, *PSMC3*, *PSMC4*, *PSMC5*, *PSMC6*, *PSMD1*, *PSMD2*, *PSMD3*, *PSMD4*, *PSMD5*, *PSMD7*, *PSMD8*, *PSMD9*, *PSMD10*, *PSMD11*, *PSMD12*, *PSMD13*, *PSME1*, *PSME2*, *RPS27A*, *LOC652826*, *RPS27AP11*, *UBA52*, *ARHGAP10*, *CASP3*, *CASP9*, *UNC5A*, *PSMF1*, *MAGED1*, *PSMD6*	0.0670
REACTOME regulation of EGFR in cancer	*AKT3*, *ADAM10*, *SPRY1*, *SPRY2*, *STAM2*, *CDKN1A*, *CDKN1B*, *ADCY1*, *ADCY2*, *ADCY3*, *ADCY5*, *CDC37*, *ADCY6*, *ADCY7*, *ADCY8*, *CHUK*, *ADCY9*, *THEM4*, *AP2M1*, *AP2S1*, *CLTA*, *CLTC*, *CREB1*, *CSK*, *ADRBK1*, *AP2A1*, *AP2A2*, *AP2B1*, *EGF*, *EGFR*, *ADCY4*, *EPS15*, *AKT1*, *AKT2*, *FOXO1*, *FOXO3*, *PHLPP1*, *MTOR*, *RICTOR*, *GAB1*, *LRIG1*, *GRB2*, *GSK3A*, *EPN1*, *SH3KBP1*, *NR4A1*, *HRAS*, *HSP90AA1*, *ITPR2*, *ITPR3*, *KRAS*, *MDM2*. *FOXO4*, *NRAS*, *PDE1A*, *PDE1B*, *PDPK1*, *PIK3CA*, *PIK3R1*, *PLCG1*, *PRKACA*, *PRKACB*, *PRKACG*, *PRKAR1A*, *PRKAR1B*, *PRKAR2A*, *PRKAR2B*, *PRKCA*, *PRKCD*, *PRKCE*, *PRKCG*, *PAG1*, *MAPK1*, *MAPK3*, *MAP2K1*, *MAP2K2*, *BAD*, *PTEN*, *TRIB3*, *EPS15L1*, *RAF1*, *RPS6KB2*, *RPS27A*, *MLST8 SH3GL2*, *SHC1*, *SOS1*, *SRC*, *ADAM17*, *TSC2*, *RPS27AP11*, *LOC729120*, *LOC730418*, *UBA52*, *LOC731292*, *YWHAB*, *MAPKAP1*, *CALM1*, *STAM*, *ADAM12*, *CALM2*, *CALM3*, *CAMK4*, *CASP9*, *AKT1S1*, *CBL*, *HGS*, *CDK1*, *CDC42*	0.9891

## Discussion

Common CNVs have been associated with predisposition to cancers such as neuroblastoma [20] and breast cancer [36]. The over-representation of common deletion genic CNVs in cases as compared to controls (Case/control ratio = 2.26, p-value = 0.03) could potentially bring about deleterious and pathogenic consequences that would lead to susceptibility to NPC. No previous studies on global CNV burden analysis were reported in NPC.

The most significantly associated CNVR from our study was within the metabotropic glutamate receptor 5 (*GRM5*) gene, suggesting its potential role in NPC pathogenesis. *GRM5* gene (approximately 563kbp) located on the minus strand of chromosome 11q14.3 encodes a group I Gq-coupled receptor. Alternative 5' splicing and usage of multiple promoters were involved in the regulatory mechanisms of GRM5 expression [37]. Evidence from studies suggests a role for glutamatergic signalling in the biology of cancer in peripheral tissues [38, 39], especially those of mucosal nature. Different groups have also shown evidence for a role of glutamate and its receptors in regulation of tumour growth [40]. Examples of metabotropic glutamate receptors being implicated in development of cancers are the involvement of GRM1 [41] and GRM5 [42] in the induction of melanoma in transgenic mice. In addition, GRM5 was found to play a role in tumour cell migration and invasion in oral squamous cell carcinoma [43] and found to be overexpressed in lung cancer cells [44].

Deletion on chromosome 6p21.3 which overlaps MHC Class I Polypeptide-Related Sequence A (*MICA)*, HLA complex 5 (*HCP5*) and HLA complex group 26 *(HCG26*) genes was previously associated to NPC predisposition. *MICA* encodes a ligand for the natural killer-cell receptor NKG2-D type II. MICA is highly expressed on cancer cells and can activate antitumor effects from natural killer cells and CD8^+^ T cells [45]. Meanwhile the function of HLA complex P5, *HCP5* is not fully understood.

The *MICA/HCP5* region has been linked to NPC susceptibility and HCV-associated hepatocellular carcinoma [14, 46]. *MICA-STR* has been implicated in NPC predisposition among male Southern Chinese Han population [47]. The frequency of the deletion in our results from combined Chinese cases and controls was 0.02, similar to the frequency reported by Tse *et al*., 2011. In addition, we also found similar results in which deleted *MICA* gene was frequently found in the *HLA-B48* (B*4801) associated haplotype in a Japanese study cohort [48]. They found 62.5% (5/8) of their samples with homozygous HLA-B*4801 allele lack intact *MICA* gene while we found 53.55% (8/15) of our samples with single copy of HLA-B*4801 allele (HLA allele imputation results) had one copy number for *MICA* gene. The concordance rate between the results from *HLA* allele imputation software and previous genotyping results of *HLA-A* allele in our laboratory [49] was 98.52%.

CNVs identified from our study were enriched for ERK1/2 and Notch signalling pathways. Mutations in GRM5 influenced Ca^2+^ oscillations in transgenic mice that showed tumour/melanoma phenotype in addition to dramatic increase in phosphorylation of ERK in these tumour samples. They had implicated ERK as a downstream effector of GRM5 signalling in tumours. The possible role of ERK pathway in NPC pathogenesis had also been corroborated by Lan *et al*. [50]. Further studies are needed to better understand the dynamics and interaction of GRM5 within the ERK pathway and its role in NPC.

We report the association of CNV 11q14.3 and 6p21.3 as well as the ERK1/2 and Notch signalling pathways with NPC susceptibility in the Malaysian Chinese cohort using a case control genome-wide SNP microarray approach. The caveat attached to SNP microarray CNV detection remains the diverse algorithmic calling methods, resulting in considerable variation in the CNV calls. We have employed extensive validation and replication using Taqman real-time PCR and a further more sensitive digital PCR method to negate false CNV calls for CNVs 11q14.3 and 6p21.3. Our study was able to detect association of CNV 11q14.3 and 6p21.3 in the Malaysian Chinese cohort but not in the Malaysian Malay cohort. However power analysis showed that power for CNV study for Malay replication cohort at 11q14.3 and 6p21.3 is 0.26 and 0.11 respectively, hence a larger sample size would be needed to improve the achieved power for better confidence. To increase reproducibility and confidence in our association, replication efforts with new NPC cohorts will aid to confirm the link between CNV 11q14.3 and 6p21.3 and NPC.

## Supporting Information

S1 Table Basic characteristics of Malaysian Chinese NPC patients and healthy controls in the study. (DOCX)

S2 Table Basic characteristics of Malaysian Malay NPC patients and healthy controls in the study. (DOCX)

S1 Text Log R ratio and B allele frequency of SNPs involved in CNVR on chromosome 6. (TXT)

S2 Text Log R ratio and B allele frequency of SNPs involved in CNVR on chromosome 11. (TXT)
