# Supplementary material for: A Genome Wide Study of Copy Number Variation Associated with Nasopharyngeal Carcinoma in Malaysian Chinese Identifies CNVs at 11q14.3 and 6p21.3 as Candidate Loci
Source: PLoS One. 2016 Jan 5;11(1):e0145774. doi: 10.1371/journal.pone.0145774 (PMC4701378; doi:10.1371/journal.pone.0145774)
Supplement: S1 Table — (DOCX) [file pone.0145774.s001.docx]

S1 Table: Basic characteristics of Malaysian Chinese NPC patients and healthy controls in the study.

| **Characteristics** | **Genome wide genotyping cohort** | | **Malaysian Chinese replication cohort** | |
| --- | --- | --- | --- | --- |
|  | Cases (n=140) | Control (n=256) | Cases (n=465) | Control (n=677) |
| **Gender**  **Male**  **Female** | 111  29 | 186  70 | 322  143 | 404  273 |
| **Age at diagnosis**  **Mean± SD (Years)**  **Range (Years)** | 53.16±9.92  29-78 | 38.19±8.68  22-59 | 54.76±9.76  19-73 | 37.05±8.23  21-67 |
