# Supplementary material for: A Genome Wide Study of Copy Number Variation Associated with Nasopharyngeal Carcinoma in Malaysian Chinese Identifies CNVs at 11q14.3 and 6p21.3 as Candidate Loci
Source: PLoS One. 2016 Jan 5;11(1):e0145774. doi: 10.1371/journal.pone.0145774 (PMC4701378; doi:10.1371/journal.pone.0145774)
Supplement: S2 Table — (DOCX) [file pone.0145774.s002.docx]

S2 Table: Basic characteristics of Malaysian Malay NPC patients and healthy controls in the study.

| **Characteristics** | **Malaysian Malay Replication cohort** | |
| --- | --- | --- |
|  | Cases (n=114) | Control (n=124) |
| **Gender**  **Male**  **Female** | 83  31 | 83  41 |
| **Age at diagnosis**  **Mean± SD (Years)**  **Range (Years)** | 49.43±14.37  14-80 | 35.60±8.49  23-60 |
